# Dietary antioxidants impact DDT resistance in *Drosophila melanogaster*

**DOI:** 10.1371/journal.pone.0237986

**Published:** 2020-08-25

**Authors:** Gamal A. M. Abdu-Allah, Keon Mook Seong, Omprakash Mittapalli, James Adebayo Ojo, Weilin Sun, Omar Posos-Parra, David Mota-Sanchez, John M. Clark, Barry R. Pittendrigh

**Affiliations:** 1 Department of Entomology, Michigan State University, East Lansing, MI, United States of America; 2 Department of Plant Protection, Assiut University, Assiut, Egypt; 3 Department of Applied Biology, Kyungpook National University, Sangju, Gyeongbuk, Republic of Korea; 4 Department of Entomology, University of Kentucky, Lexington, KY, United States of America; 5 Department of Crop Production, Kwara State University, Malete, Ilorin, Nigeria; 6 Department of Veterinary and Animal Sciences, University of Massachusetts, Amherst, MA, United States of America; University of Crete, GREECE

## Abstract

Insects experience a diversity of subtoxic and/or toxic xenobiotics through exposure to pesticides and, in the case of herbivorous insects, through plant defensive compounds in their diets. Many insects are also concurrently exposed to antioxidants in their diets. The impact of dietary antioxidants on the toxicity of xenobiotics in insects is not well understood, in part due to the challenge of developing appropriate systems in which doses and exposure times (of both the antioxidants and the xenobiotics) can be controlled and outcomes can be easily measured. However, in *Drosophila melanogaster*, a well-established insect model system, both dietary factors and pesticide exposure can be easily controlled. Additionally, the mode of action and xenobiotic metabolism of dichlorodiphenyltrichloroethane (DDT), a highly persistent neurotoxic organochlorine insecticide that is detected widely in the environment, have been well studied in DDT-susceptible and -resistant strains. Using a glass-vial bioassay system with blue diet as the food source, seven compounds with known antioxidant effects (ascorbic acid, β-carotene, glutathione, α-lipoic acid, melatonin, minocycline, and serotonin) were orally tested for their impact on DDT toxicity across three strains of *D*. *melanogaster*: one highly susceptible to DDT (*Canton-S*), one mildly susceptible (*91-C*), and one highly resistant (*91-R*). Three of the antioxidants (serotonin, ascorbic acid, and β-carotene) significantly impacted the toxicity of DDT in one or more strains. Fly strain and gender, antioxidant type, and antioxidant dose all affected the relative toxicity of DDT. Our work demonstrates that dietary antioxidants can potentially alter the toxicity of a xenobiotic in an insect population.

## Introduction

Pesticide resistance is an ongoing problem for those tasked with pest management [1]. Resistance can occur through multiple mechanisms including target site insensitivity, reduced penetration, and sequestration as well as through changes in phase I, II, or III detoxification enzymes and transporters [2–6]. The chemical composition of the active toxin, including the pesticide class, level of exposure, duration of selection, genetic variation of the target species, and possibly other biotic and abiotic factors may also play roles in the evolution of resistance [1]. One well-studied pesticide insect model has been DDT (dichlorodiphenyltrichloroethane) resistance in the fruit fly, *Drosophila melanogaster*, which has served as a model organism for more than 70 years. *D*. *melanogaster* is easy to rear; several strains are available to use; its diet can be tightly controlled, which allows exposure to known levels of xenobiotics over defined periods of time; and there is a bioassay system with which toxicity outcomes can be easily measured [7, 8]. *D*. *melanogaster* is also a genetically tractable system, with a considerable number of molecular tools that have allowed the molecular basis of DDT resistance to be studied in detail [9–12].

DDT is an effective oxidative stressor, and its toxicity and cellular impacts associated with oxidative stress have been demonstrated through biomarkers known to produce free radicals that damage DNA [13, 14]. Lipid peroxidation, activation of free radicals, and changes in antioxidant enzymes and in the glutathione redox system are known indicators of oxidative stress in cells and tissues [15]. DNA fragmentation and cell death mediated by oxidative stress occur in the testes of male rats exposed to subacute levels of DDT [16]. Previous studies of hepatic oxidative stress markers revealed significant increases in lipid peroxide and 8-hydroxydeoxyguanosine in DDT-treated rats that developed hepatocellular tumors [17]. Increases in 8-hydroxydeoxyguanosine levels are thought to increase oxidative DNA damage and contribute to hepatocarcinogenesis in DDT-exposed rats [17]. Thus, DDT application may enhance overproduction of reactive oxygen species (ROS), leading to an imbalance in the production of free radicals and cellular antioxidants [18]. Many herbivorous insects consume antioxidants in their plant-based diets. Vitamins A, C, and E and carotenoids are some of the common environmental antioxidants obtained from plant-based diets [19]. Antioxidants and their related bioactive substances in plants and food supplements have the potential to alleviate pesticide-induced damage [20]. However, little work has been done to understand how dietary antioxidants may influence the toxicity of pesticides in insect populations.

Here we used the well-established *D*. *melanogaster* DDT bioassay system to characterize the impact of a series of dietary antioxidants on DDT resistance [21]. The objective of this study was to learn not only how seven different antioxidants (ascorbic acid, β-carotene, glutathione, α-lipoic acid, melatonin, and minocycline and serotonin [also a neurotransmitter]) impacted the resistance levels of *D*. *melanogaster* to DDT but also whether gender and genotype influenced the efficacy of antioxidants in modifying DDT toxicity.

## Materials and methods

### D. melanogaster strains

The DDT-susceptible strain *Canton-S* (originally obtained from the Bloomington Drosophila Stock Center, Bloomington, IN), the mildly DDT-resistant strain *91-C*, and the highly DDT-resistant *91-R* strain of *D*. *melanogaster* have been maintained in the Pittendrigh laboratory for almost two decades and reared at ~25°C as previously described by Strycharz et al. [6] and Seong et al. [22].

### Chemicals used in this study

The insecticide DDT (≥98% pure); the antioxidant reagents ascorbic acid (vitamin C), β-carotene (provitamin A), glutathione, α-lipoic acid, melatonin, minocycline hydrochloride, and serotonin; and the solvents (acetone and ethanol) were all purchased from Sigma-Aldrich (St. Louis, MO, USA). All chemicals were of analytical grade.

### Antioxidant supplementation

Blue diet (Formula 4–24^®^) was purchased from Carolina Biological Supply Company (Burlington, NC, USA). Before use, 1 g of blue diet was dissolved in 3.5 ml of distilled water in a 20-ml vial. For the antioxidant-supplemented treatments, this same amount of blue diet was dissolved in freshly prepared antioxidant solution. The concentrations of test antioxidants were chosen based on levels used in previous studies [23, 24]. Solutions were prepared of ascorbic acid (0.36 mM), β-carotene (0.5 μM), glutathione (0.13 mM), α-lipoic acid (1.08 mM), melatonin (0.43 mM), minocycline hydrochloride (0.05 mM), and serotonin, which was tested at an initial concentration of 860 μM followed by three additional concentrations of 0.86, 8.6, and 86 μM due to the significant response to the initial concentration (see Results). Fresh solutions were prepared on the day of use. All antioxidants were dissolved in distilled water except β-carotene, α-lipoic acid, and melatonin, which were first dissolved in ethanol (50 mg/ml; 100% [molecular biology grade]) and then diluted in distilled water. For each tested combination of fly strain by sex, about 50–70 adult flies (1–3 days old) were transferred to 20-ml vials containing antioxidant-spiked diet or control diet (no antioxidant). The vials were capped with cotton plugs and the flies were allowed to feed on the antioxidant or control diet for 5 days. The antioxidant-treated flies and controls were reared on the same shelf under the natural daylight of a North American summer. The flies were tested for DDT toxicity after 5 days of feeding.

### DDT acute toxicity bioassay

The DDT acute toxicity assay was conducted according to the method of Seong et al. [25] with slight modification: 200-μl aliquots of different dilutions of DDT stock (250 mg/ml) solutions prepared in acetone were added to individual 20-ml glass scintillation vials in three replicates. To coat the inside wall of the vials uniformly with DDT, the vials were rolled on a hot dog roller with no heat until the acetone evaporated. For the control, 200 μl of acetone was used to coat the vials. After 5 days of feeding on antioxidant-spiked diet or control diet, flies were sorted by sex into vials coated with DDT or acetone (control). Ten flies (6–8 days old) per replicate (3 replicates per DDT concentration) were used in the DDT test. DDT concentrations for *Canton-S* without antioxidants were 0.25, 0.50, 1.00, 1.50, 2.00, 10.00, and 25.00 μg/vial; with antioxidants were 0.05, 0.10, 0.25, 0.50, 1.00, 2.50, 5.00, and 20.00 μg/vial. For *91-C*, DDT concentrations without antioxidants were 2.00, 4.00, 5.00, 8.00, 10.00, 16.00, 25.00, and 50.00 μg/vial; with antioxidants were 0.10, 0.50, 1.00, 2.50, 5.00, 10.00, 25.00, 100.00, and 250.00 μg/vial. For *91-R*, DDT concentrations without antioxidants were 25.00, 50.00, 500.00, 2500.00, 5000.00, and 10000.00 μg/vial; with antioxidants were 100.00, 500.00, 1000.00, 2500.00, 5000.00, 10000.00, and 50000.00 μg/vial. Survivorship was evaluated after 24 h at room temperature. The flies were considered dead when there was no longer any movement or leg twitching after probing. For each antioxidant treatment, all data were collected on the same day.

Percent mortality was determined by probit analysis using SPSS software (Version 16.0 for Windows; SPSS Inc., Chicago, IL, USA). The same software was used to estimate the LC_50_, the 95% confidence limit (CL) values, the slope, and the χ^2^. LC_50_ values were considered significantly different when their 95% CLs did not overlap [26]. Log concentration versus percent mortality (probit) regression lines were generated to determine the DDT concentration that killed 50% of the flies (LC_50_) from each treatment.

## Results

### DDT resistance profile of D. melanogaster strains

The toxicities of DDT following a 24 h contact exposure using the vial bioassay of the *Canton-S*, *91-C*, and *91-R* strains of *D*. *melanogaster* are given in Table 1 and S1 Fig. The *Canton-S* strain was the most susceptible to DDT, with LC_50_ values of 4.32 and 0.87 μg/vial DDT for females and males, respectively. The *91-C* strain was significantly more resistant to DDT than *Canton-S* when compared within each sex, with LC_50_ values of 9.65 and 3.35 μg/vial DDT for females and males, respectively. The *91-R* strain was the most resistant to DDT, with LC_50_ values of 1857.04 and 1671.04 μg/vial DDT for females and males, respectively.

**Table 1:** LC_50_ (μg/vial DDT) values with 95% confidence limits (CL), slope, resistance ratio (RR), and gender effect (GE) for *D*. *melanogaster Canton-S*, *91-C*, and *91-R* adult females and males fed on diet without antioxidants.

Strain	Sex	LC_50_^a^ (95% CL)	Slope ±SE	RR^b^	GE^c^
***Canton-S***	♀	4.32^**e**^ (2.48–6.32)	1.68±0.27	1.00	4.97
	♂	0.87^**d**^ (0.41–1.69)	3.33±0.53	1.00	
***91-C***	♀	9.65^**f**^ (6.90–15.79)	1.36±0.36	2.23	2.88
	♂	3.35^**e**^ (1.75–4.73)	2.58±0.94	3.85	
***91-R***	♀	1857.04^**g**^ (1346.60–2223.45)	1.78±0.44	429.87	1.11
	♂	1671.04^**g**^ (1263.30–2070.95)	1.68±0.44	1920.74	

^a^ LC_50_ [Lethal concentration (μg/vial DDT) that killed 50% of the flies].

^b^ Resistance ratio (RR) = LC_50_ of the tested strain / LC_50_ of the *Canton-S* strain of the same gender.

^c^ Gender effect (GE) = Female LC_50_ / Male LC_50_ of the same strain.

^d,e,f,g^ LC_50_ values marked with different lower-case letters are significantly different based on non-overlap of 95% confidence limits.

In the absence of antioxidant treatment, the resistance ratios (RRs; LC_50_ of tested strain / LC_50_ of *Canton-S*) for *91-R* were 430 and 1921 for females and males, respectively, and 2.23 and 3.85 for *91-C* females and males, respectively. Interestingly, the highest slope value was obtained for *Canton-S* males (3.33), and the lowest slope was for *91-C* females (1.36), indicating that the difference between LC_10_ and LC_90_ was largest for *Canton-S* males. Females were more resistant than males in two of the three strains, with the female LC_50_ value being significantly higher than the male value in *Canton-S* and *91-C*, while there was no significant difference between females and males of *91-R*. The gender effect (GE) values (female LC_50_ / male LC_50_) were 4.97, 2.88, and 1.11 for *Canton-S*, *91-C*, and *91-R*, respectively. The GE reflects the body size, food intake and metabolism difference of female and male flies in these strains. These results are the basis for comparison with the effects of antioxidants described in the following sections.

### Impact of antioxidants on DDT resistance

**Fig 1:**
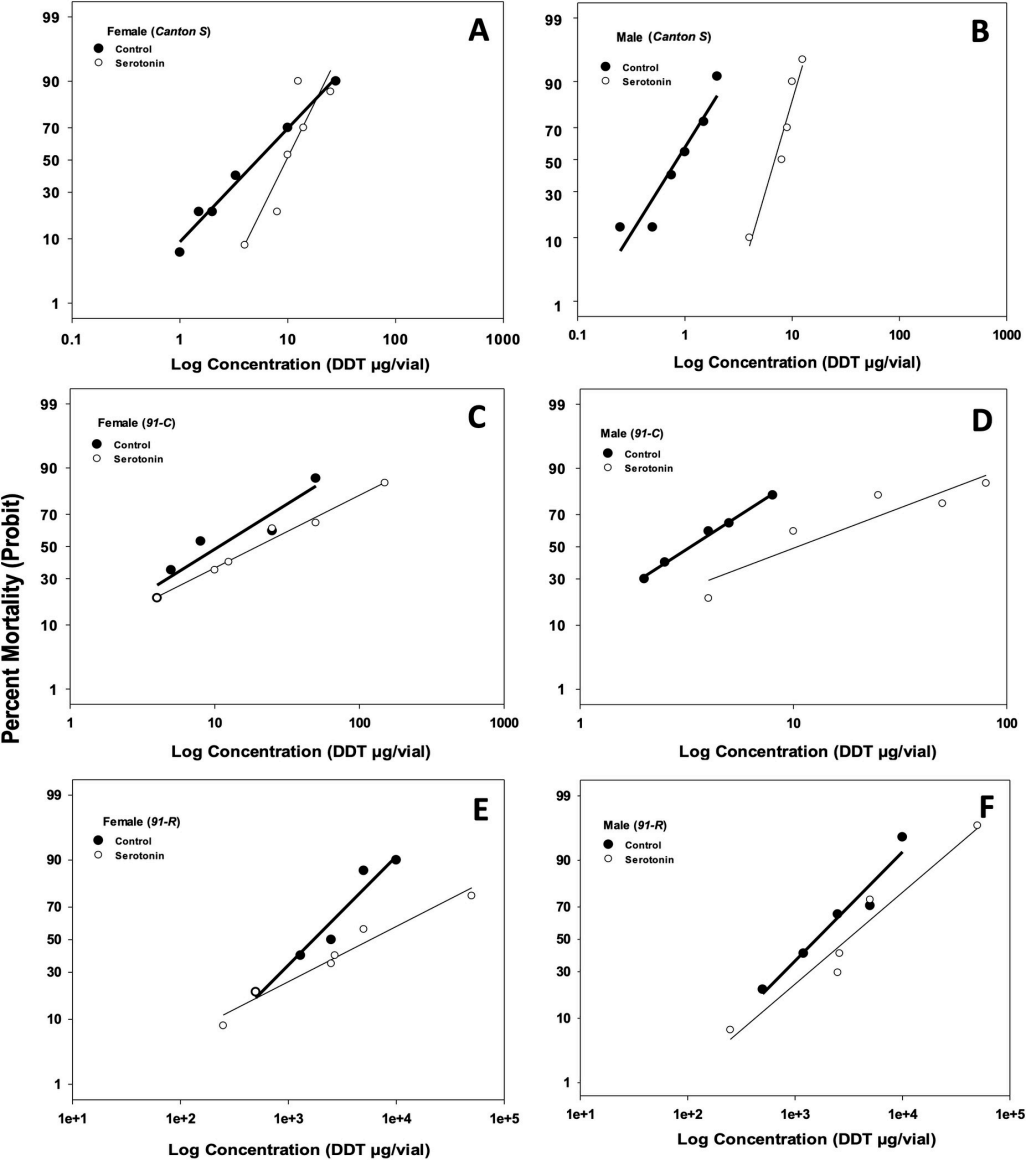
Dose response curves for DDT toxicity for females and males of *Canton-S* (A and B), *91-C* (C and D), and *91-R* (E and F) strains of *Drosophila melanogaster* raised on blue diet containing serotonin. Adults (6–8 days old) were exposed to different doses of DDT and mortality was determined at 24 h of exposure. Data were analyzed using probit analysis in SPSS (Chicago, IL, USA). For each dose, 3–4 replicates were performed.

None of the antioxidants fed to DDT-susceptible *Canton-S* females had a significant effect on resistance to DDT except for serotonin (LC_50_ of 10.25 vs control LC_50_ of 4.32 μg/vial DDT; Table 2, Fig 1, S2 Fig). Serotonin had the highest RR (LC_50_ of antioxidant / LC_50_ of Control; 2.37), while minocycline had the lowest (0.42; Table 2, S2 Fig). In descending order, the antioxidant effectiveness based on RR was serotonin > ascorbic acid > α-lipoic acid > melatonin > glutathione > β-carotene > minocycline. *Canton-S* females showed relatively higher homogeneity in response to DDT than males, with slope values varying from 0.98 to 3.58 for females vs 1.66 to 6.69 for males (Table 2). In the case of *Canton-S* males, serotonin showed the highest RR (8.49), with an LC_50_ (7.39 μg/vial) significantly higher than that of the control (0.87 μg/vial) (Table 2). Of the remaining treatments, only ascorbic acid resulted in an RR greater than 1 (1.72), but the LC_50_ was not significantly different from that of the control (Table 2). None of the remaining antioxidants tested resulted in any significant effect when compared to control toxicity values (Table 2).

**Table 2:** LC_50_ (μg vial/DDT) values with 95% confidence limits (CL), slope, χ^2^, and resistance ratio (RR) for *D*. *melanogaster Canton-S* adult females and males fed on the indicated antioxidants.

Treatment	Female					Male				
	LC_50_ (95% CL)	Slope ±SE	χ^2^	*P*	RR^a^	LC_50_ (95% CL)	Slope ±SE	χ^2^	*P*	RR^a^
**Control**	4.32 (2.48–6.32)	1.68±0.27	14.05	0.001	1.00	0.87 (0.41–1.69)	3.33±0.53	0.18	0.914	1.00
**Ascorbic acid**	4.23 (1.37–5.08)	1.61±0.26	10.69	0.013	0.98	1.50 (1.01–2.06)	2.03±0.31	2.41	0.299	1.72
**β-Carotene**	2.44 (1.44–3.55)	1.33±0.31	14.05	0.001	0.56	0.19 (0.12–0.46)	2.67±0.60	0.51	0.774	0.22
**Glutathione**	2.61 (1.45–4.22)	2.41±0.41	6.28	0.090	0.60	0.36 (0.20–3.20)	3.46±0.75	12.14	0.033	0.41
**α-Lipoic acid**	3.45 (1.63–5.11)	0.98±0.18	5.69	0.127	0.80	0.44 (0.31–0.62)	1.75±0.26	4.84	0.303	0.51
**Melatonin**	2.88 (2.06–4.60)	2.53±0.58	0.57	0.984	0.67	0.82 (0.66–1.08)	3.62±0.60	6.69	0.153	0.94
**Minocycline**	1.81 (1.20–3.13)	1.95±0.36	10.68	0.014	0.42	0.51 (0.4–0.88)	1.66±0.25	7.76	0.051	0.59
**Serotonin**	10.25^**b**^ (6.66–13.89)	3.58±0.47	11.98	0.007	2.37	7.39^**b**^ (4.45–10.81)	6.69±1.08	8.70	0.034	8.49

^a^ Resistance ratio (RR) = LC_50_ of antioxidant / LC_50_ of Control.

^b^ LC_50_ value is significantly different from that for blue diet alone (Control) for that gender, based on non-overlap of 95% confidence limits.

With *91-C* flies (low-level DDT resistance), only serotonin resulted in significantly higher LC_50_ values than the controls for both females and males, with RRs of 2.09 and 3.20, respectively (Table 3; S3 Fig). None of the remaining treatments resulted significantly increased LC_50_ values from their controls for either female or male flies except for β-carotene, which had a significantly lower LC_50_ for both females and males (Table 3).

**Table 3:** LC_50_ (μg/vial DDT) values with 95% confidence limits (CL), slope, χ^2^, and resistance ratio (RR) for *D*. *melanogaster 91-C* adult females and males fed on the indicated antioxidants.

Treatment	Female					Male				
	LC_50_ (95% CL)	Slope ±SE	χ^2^	*P*	RR^a^	LC_50_ (95% CL)	Slope ±SE	χ^2^	*P*	RR^a^
**Control**	9.65 (6.90–15.79)	1.36±0.36	2.69	0.262	1.00	3.35 (1.75–4.73)	2.58±0.94	0.34	0.955	1.00
**Ascorbic acid**	7.85 (5.61–11.22)	2.08±0.49	3.01	0.222	0.81	2.26 (0.34–3.48)	1.96±0.70	1.89	0.169	0.67
**β-Carotene**	4.26^b^ (3.04–5.78)	1.91±0.49	0.04	0.981	0.44	1.07^b^ (0.70–1.45)	3.23±0.66	1.10	0.575	0.32
**Glutathione**	10.56 (7.11–15.35)	1.63±0.29	4.50	0.480	1.09	3.17 (1.38–5.84)	1.75±0.26	20.36	0.001	0.95
**α-Lipoic acid**	14.59 (8.72–16.66)	4.44±1.53	0.002	0.963	1.51	2.18 (1.51–3.39)	1.15±0.37	1.14	0.887	0.65
**Melatonin**	12.32 (8.83–16.78)	1.17±0.18	11.16	0.023	1.28	1.85 (1.39–2.38)	3.05±0.54	3.35	0.500	0.55
**Minocycline**	14.34 (4.49–18.94)	0.89±0.25	3.03	0.219	1.49	0.44 (0.25–1.76)	1.63±0.30	3.68	0.595	0.13
**Serotonin**	20.17^**c**^ (15.43–25.44)	1.16±0.42	0.35	0.945	2.09	10.72^**c**^ (4.79–15.65)	1.18±0.38	2.03	0.364	3.20

^a^ Resistance ratio (RR) = LC_50_ of antioxidant / LC_50_ of Control.

^**b, c**^ LC_50_ value is significantly different from that for blue diet alone (Control) for that gender, based on non-overlap of 95% confidence limits.

The results for the antioxidant treatments of female flies of the highly DDT-resistant *91-R* strain are shown in Table 4, and S4 Fig. Treatments with serotonin, ascorbic acid, and β-carotene resulted in significantly higher LC_50_ values (5652.39, 4339.08, and 4312.11 μg/vial DDT, respectively) than the control (1857.04 μg/vial DDT), with RR values of 3.04, 2.34, and 2.32, respectively. For *91-R* males, serotonin treatment resulted in a significantly higher LC_50_ value (3843.19 μg/vial DDT) than that of the control (1671.04 μg/vial), with an RR value of 2.30 (Table 4), whereas ascorbic acid and β-carotene did not have any significant effect. None of the remaining treatments resulted in LC_50_ values that were significantly different from their controls for either female or male flies.

**Table 4:** LC_50_ (μg/vial DDT) values with 95% confidence limits (CL), slope, χ^2^, and resistance ratio (RR) for *D*. *melanogaster 91-R* adult females and males fed on the indicated antioxidants.

Treatment	Female					Male				
	LC_50_ (95% CL)	Slope ±SE	χ^2^	*P*	RR^a^	LC_50_ (95% CL)	Slope ±SE	χ^2^	*P*	RR^a^
**Control**	1857.04 (1346.60–2223.45)	1.78±0.44	1.52	0.466	1.00	1671.04 (1263.30–2070.95)	1.68±0.44	1.52	0.463	1.00
**Ascorbic acid**	4339.08^**b**^ (2500.23–5600.44)	0.91±0.31	11.62	0.003	2.34	2848.33 (2046.33–4048.72)	1.78±0.39	1.98	0.372	1.70
**β-Carotene**	4312.11^**b**^ (2418.36–5521.79)	1.25±0.52	1.68	0.197	2.32	1792.65 (1300.13–2175.59)	0.53±0.43	0.07	0.781	1.07
**Glutathione**	1797.45 (730.84–1807.31)	0.68±0.24	5.03	0.165	0.97	1257.34 (1162.17–1511.47)	1.37±0.27	0.81	0.844	0.75
**α-Lipoic acid**	2441.39 (1774.57–2910.28)	1.85±0.53	12.37	0.001	1.31	1283.46 (950.91–1947.85)	1.46±0.26	30.87	0.001	0.77
**Melatonin**	2148.03 (1962.12–2839.70)	1.94±0.35	6.98	0.072	1.16	1023.38 (860.12–2078.03)	1.64±0.41	4.13	0.247	0.61
**Minocycline**	1561.25 (956.70–2484.90)	1.01±0.26	3.75	0.289	0.84	1094.36 (753.72–1472.21)	1.05±0.19	4.06	0.540	0.65
**Serotonin**	5652.39^**b**^ (3650.55–7046.09)	0.81±0.12	1.34	0.714	3.04	3843.19^**b**^ (2372.67–6239.98)	1.61±0.29	3.79	0.154	2.30

^a^ Resistance ratio (RR) = LC_50_ of antioxidant / LC_50_ of Control.

^b^ LC_50_ value is significantly different from that for blue diet alone (Control) for that gender, based on non-overlap of 95% confidence limits.

### Effect of increasing concentrations of serotonin on DDT toxicity in Canton-S flies

The effects of varying concentrations of serotonin on DDT mortality of female *Canton-S* flies are shown in S1 Table and S5 Fig. In the single dose experiment (Table 2), serotonin at 860.00 μM significantly increased the DDT LC_50_ value (10.25 μg/vial DDT) compared to the control (4.32 μg/vial DDT), with an RR value of 2.37 for females. Similarly, serotonin at 860.00 μM also significantly increased the DDT LC_50_ value for *Canton-S* male flies (7.39 μg/vial DDT) compared to the control (0.87 μg/vial DDT), with an RR value of 8.49 (Table 2). Interestingly, all three lower concentrations of serotonin (0.86, 8.60, and 86.00 μM) actually increased DDT toxicity in female flies (S1 Table), with the 8.60 and 86.00 μM concentrations causing significant reductions in LC_50_ compared with the controls for both female and male flies.

## Discussion

A number of defense mechanisms in insect species help diminish the levels of oxidative damage induced by insecticides. In this study, three *Drosophila* strains with varying levels of DDT resistance (*Canton-S*, *91-C*, and *91-R*) were tested for the effects of dietary antioxidants. Serotonin at 860 μM increased the level of DDT resistance in all three strains, both male and female, based on LC_50_ values. In addition, β-carotene and ascorbic acid had significant increased DDT resistance in the *91-R* female flies, whereas the effects of α-lipoic acid, melatonin, glutathione, and minocycline hydrochloride were insignificant in all three strains, regardless of gender. Thus, the significant increase in DDT resistance of flies treated with serotonin, and in some cases with β-carotene or ascorbic acid, could be a result of their antioxidant and scavenger properties against oxidant molecules induced by DDT.

Although the antioxidative properties of serotonin are less documented than those of the other compounds tested here, serotonin showed an antioxidative effect at 3- to 10-fold lower concentrations than melatonin in a previous study [27]. Serotonin also exhibited antioxidant and membrane-binding properties that protect lipids from oxidation when it was added to red blood cells and lipid membranes with different levels of unsaturation [28]. Serotonin could be functioning in a similar manner in *D*. *melanogaster* exposed to DDT: specifically, the external source of serotonin may function in scavenging the free radicals produced in response to DDT.

In addition to having antioxidant properties, serotonin is a neurotransmitter [29]. Given that DDT primarily targets the neuromuscular junction, resulting in accelerated spontaneous release of neurotransmitters, this mode of action of DDT could lead to depletion of neurotransmitters [30]. Therefore, we propose that dietary intake of serotonin may help supplement the DDT-induced loss of neurotransmitters, thereby providing a higher tolerance level in *D*. *melanogaster*. Serotonin is an inhibitory neurotransmitter that binds to 5-hydroxytryptamine receptors and glutamate-gated chloride channels, which then open up and hyperpolarize the cell [31]. This effect of serotonin is opposite that of DDT, providing a plausible explanation for how serotonin increases tolerance to DDT’s neurotoxic effects. However, serotonin’s functional roles as a neurotransmitter and as an effective antioxidant remain to be further elucidated in *D*. *melanogaster*.

Both β-carotene and ascorbic acid have been extensively studied regarding how their antioxidant activities protect DNA from oxidative free radical damage [32, 33]. Analogously, Cozzi et al. [34] proposed that high dietary ascorbic acid is considered an efficient antioxidant via its action as a modulator of oxidative damage. One hypothesis for the protective effects of ascorbic acid and β-carotene on *91-R*, but not on the other two strains, is that the *91-R* strain is known to have greater levels of cuticular hydrocarbons and oxidative metabolism of DDT to dicofol using cytochrome P450 monooxygenases [6]; thus, one would expect increased ROS levels due to increased P450 activity in this strain. If so, perhaps the *91-R* flies are under more oxidative stress and hence are protected by powerful antioxidants, *i*.*e*., ascorbic acid and β-carotene, in addition to serotonin. This hypothesis remains to be tested.

In a previous study, β-carotene and fucoxanthin enhanced extension of the flies’ lifespan, reduced mortality of female flies, and enhanced resistance of female *D*. *melanogaster* to paraquat oxidative stress [35]. Notably, the effects of β-carotene and fucoxanthin on lifespan and expression of stress response genes were more pronounced in females than in males, which could be attributed to the physiological differences between sexes and to females consuming more food during their lifespan [36]. In the current study, β-carotene significantly increased tolerance to DDT exposure in *91-R* females as compared to non-treated control females. This result may suggest that β-carotene affects stress response gene expression involved in insecticide detoxification in *D*. *melanogaster*. Additionally, β-carotene plays a protective role in the antioxidant defense mechanism against imidacloprid in albino rats [37]. Also, el-Demerdash et al. [38] reported that β-carotene decreased the levels of harmful free radicals produced by the pyrethroid insecticide fenvalerate in rats. Therefore, the augmentative effect of β-carotene on development of tolerance against DDT toxicity may be due to its role as an antioxidant through quenching of ROS, this remains to be tested in the future studies.

Ascorbic acid is a primary antioxidant found in insects [39], and our investigation supports earlier studies of ascorbic acid performing the role of scavenger [40, 41]. The antioxidant vitamins C and E provided pretreated erythrocytes with significant protection against the cytotoxic effects of dimethoate-induced oxidative stress by decreasing lipid peroxidation via the induction of cellular antioxidant enzymes superoxide dismutase (SOD) and catalase (CAT) [42]. The combination of ascorbic acid and E alleviated the harmful effects of copper by decreasing lipid peroxidation and hepatic enzymes in broiler chickens [43]. In insects, dietary studies have demonstrated that ascorbic acid supplementation affects the aging process, extending the average life span of wild-type *D*. *melanogaster* [40]. Furthermore, ascorbic acid increased the activation of glutathione-S-transferases (GSTs), which plays an important role in converting DDT to nontoxic dichlorodiphenyldichloroethylene (DDE), in honey bees [41]. Therefore, the effects of ascorbic acid on DDT resistance level in this study may suggest that the dietary source of antioxidant ascorbic acid triggers the induction of internal antioxidant enzymes such as SOD, CAT, and GSTs that ultimately contribute to the defense response to DDT in *D*. *melanogaster* strains. However, additional analysis will likely be required to investigate the enzyme activity changes in response to antioxidant exposure, so this hypothesis has to be taken with caution. Indeed, a number of studies have revealed that low-molecular-weight antioxidants such as vitamin C and E exert protective effects against oxidative stress [44]. In this study, both serotonin and vitamin C increased resistance to DDT in *91-R*, whereas the effect of minocycline was insignificant in all three *D*. *melanogaster* strains. These results possibly indicate that the low molecular weights of serotonin and ascorbic acid (176.21 and 176.12 g/mol, respectively), as compared to minocycline hydrochloride (457.48 g/mol), may result in higher tolerance to oxidative stress induced by DDT.

## Conclusion

Our results hold out the possibility that increases in antioxidants in the diet may have the potential to impact resistance to pesticides. This hypothesis needs to be tested across pesticide classes, species, and dietary levels of antioxidants to better understand this relationship. The most practical impacts of these outcomes may come in understanding the impacts of dietary antioxidants that insects may experience in their diets (e.g., *D*. *suzukii* living on blueberries) in relationship to the evolution of resistance. Recently, Huang and co-workers [45] demonstrated that dietary ascorbic acid can influence the evolution of DDT resistance in *D*. *melanogaster*. The impact that other dietary antioxidants have on multi-generational selection by pesticides and the evolution of tolerance/resistance, if any, remains to be determined.

## Supporting information

S1 Fig LC_50_ (μg/vial DDT) values with 95% confidence limits for adult females and males of *D*. *melanogaster* strains *Canton-S*, *91-C*, and *91-R* without antioxidant treatment. LC_50_ values marked with different lower-case letters are significantly different based on non-overlap of 95% confidence limits.
(PNG)

S2 Fig Dose response curves for DDT toxicity for females (A, B) and males (C, D) of *D*. *melanogaster* strain *Canton-S* fed on blue diet plus antioxidants (ascorbic acid, β-carotene, glutathione, α-lipoic acid, melatonin, minocycline hydrochloride, serotonin). Adults (6–8 days old) were exposed to different doses of DDT and mortality was determined 24 h after exposure. Data were analyzed using probit analysis in SPSS (Chicago, IL, USA). For each dose, 3–4 replicates were conducted.
(PNG)

S3 Fig Dose response curves for DDT toxicity for females (A, B) and males (C, D) of *D*. *melanogaster* strain *91-C* fed on blue diet plus antioxidants (ascorbic acid, β-carotene, glutathione, α-lipoic acid, melatonin, minocycline hydrochloride, serotonin). Adults (6–8 days old) were exposed to different doses of DDT and mortality was determined 24 h after exposure. Data were analyzed using probit analysis in SPSS (Chicago, IL, USA). For each dose, 3–4 replicates were conducted.
(PNG)

S4 Fig Dose response curves for DDT toxicity for females (A, B) and males (C, D) of *D*. *melanogaster* strain *91-R* fed on blue diet plus antioxidants (ascorbic acid, β-carotene, glutathione, α-lipoic acid, melatonin, minocycline hydrochloride, serotonin). Adults (6–8 days old) were exposed to different doses of DDT and mortality was determined 24 h after exposure. Data were analyzed using probit analysis in SPSS (Chicago, IL, USA). For each dose, 3–4 replicates were conducted.
(PNG)

S5 Fig Dose response curves for DDT toxicity for females (A) and males (B) of *D*. *melanogaster* strain *Canton-S* fed on blue diet plus different doses of serotonin. Adults (6–8 days old) were exposed to different doses of DDT and mortality was determined 24 h after exposure. Data were analyzed using probit analysis in SPSS (Chicago, IL, USA). For each dose, 3–4 replicates were conducted.
(PNG)

S1 Table LC_50_ (μg/vial DDT) values with 95% confidence limits (CL), slope, χ^2^, and resistance ratio (RR) for *D*. *melanogaster Canton-S* adult females and males fed on the indicated serotonin doses. (DOCX)
