# Supplementary figures and images for: Dietary antioxidants impact DDT resistance in Drosophila melanogaster
Source: PLoS One. 2020 Aug 25;15(8):e0237986. doi: 10.1371/journal.pone.0237986 (PMC7447025; doi:10.1371/journal.pone.0237986)

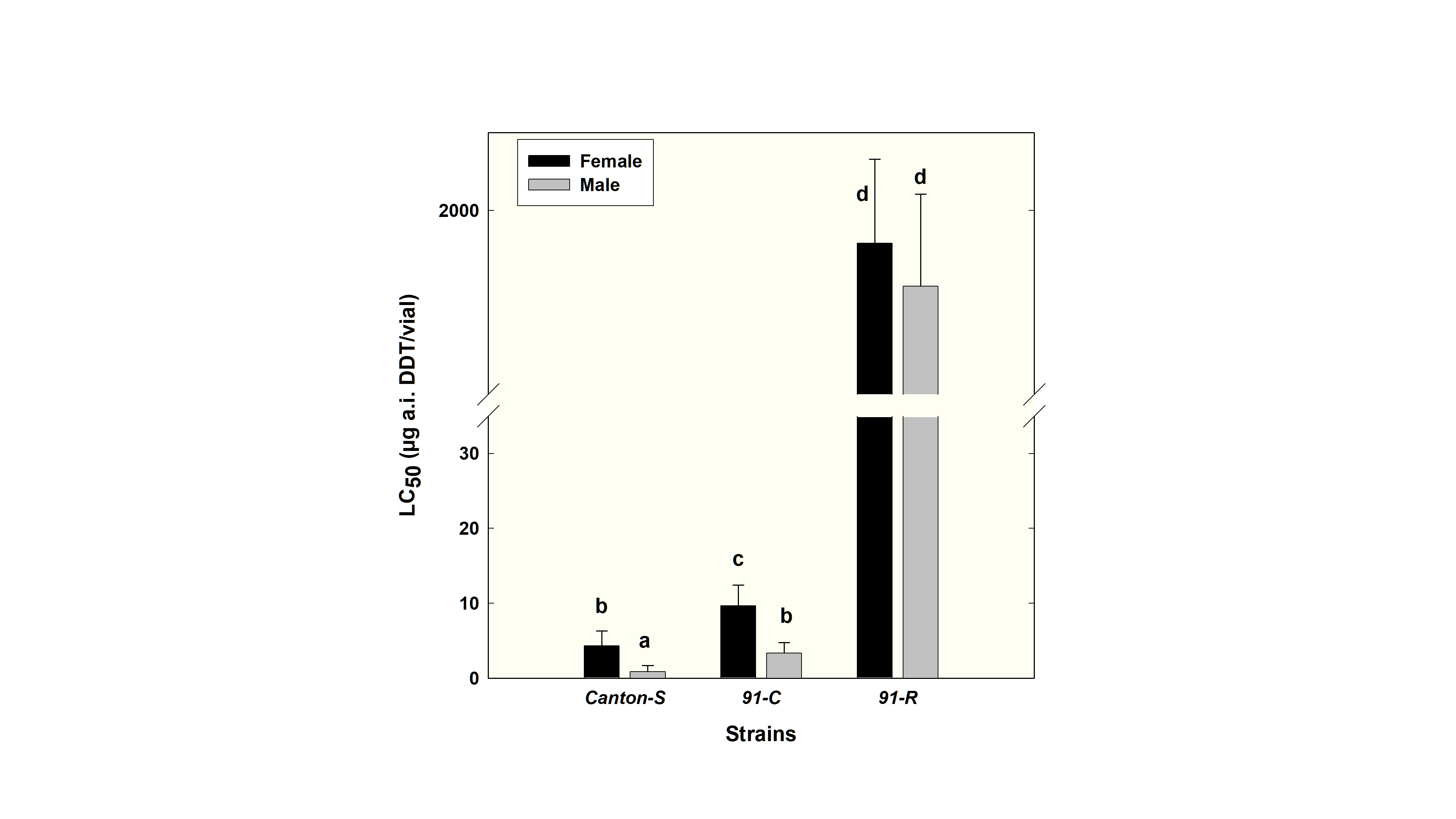

Supplement: S1 Fig — LC50 values marked with different lower-case letters are significantly different based on non-overlap of 95% confidence limits. (PNG) [file pone.0237986.s001.png]

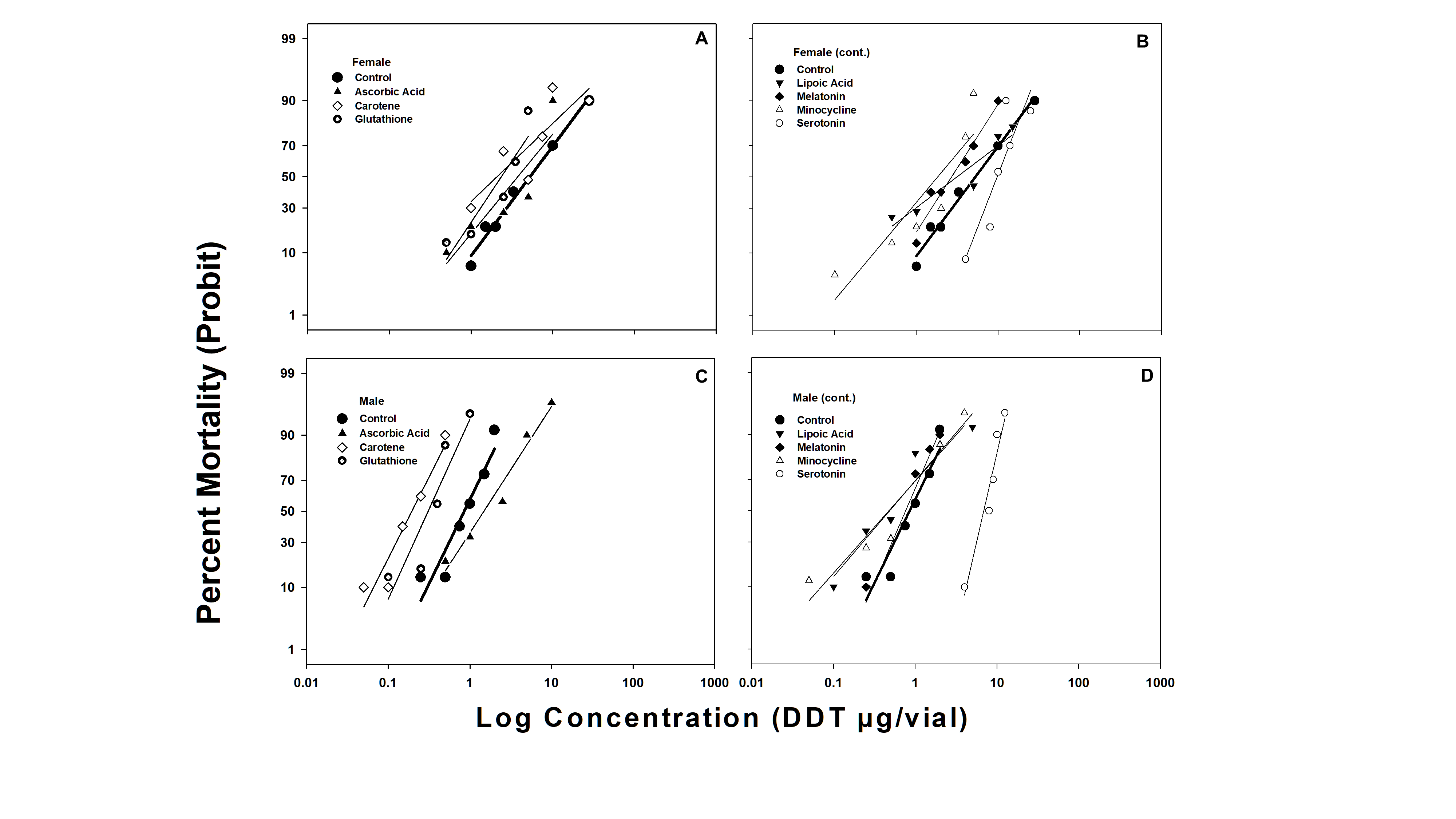

Supplement: S2 Fig — Dose response curves for DDT toxicity for females (A, B) and males (C, D) of D. melanogaster strain Canton-S fed on blue diet plus antioxidants (ascorbic acid, β-carotene, glutathione, α-lipoic acid, melatonin, minocycline hydrochloride, serotonin). Adults (6–8 days old) were exposed to different doses of DDT and mortality was determined 24 h after exposure. Data were analyzed using probit analysis in SPSS (Chicago, IL, USA). For each dose, 3–4 replicates were conducted. (PNG) [file pone.0237986.s002.png]

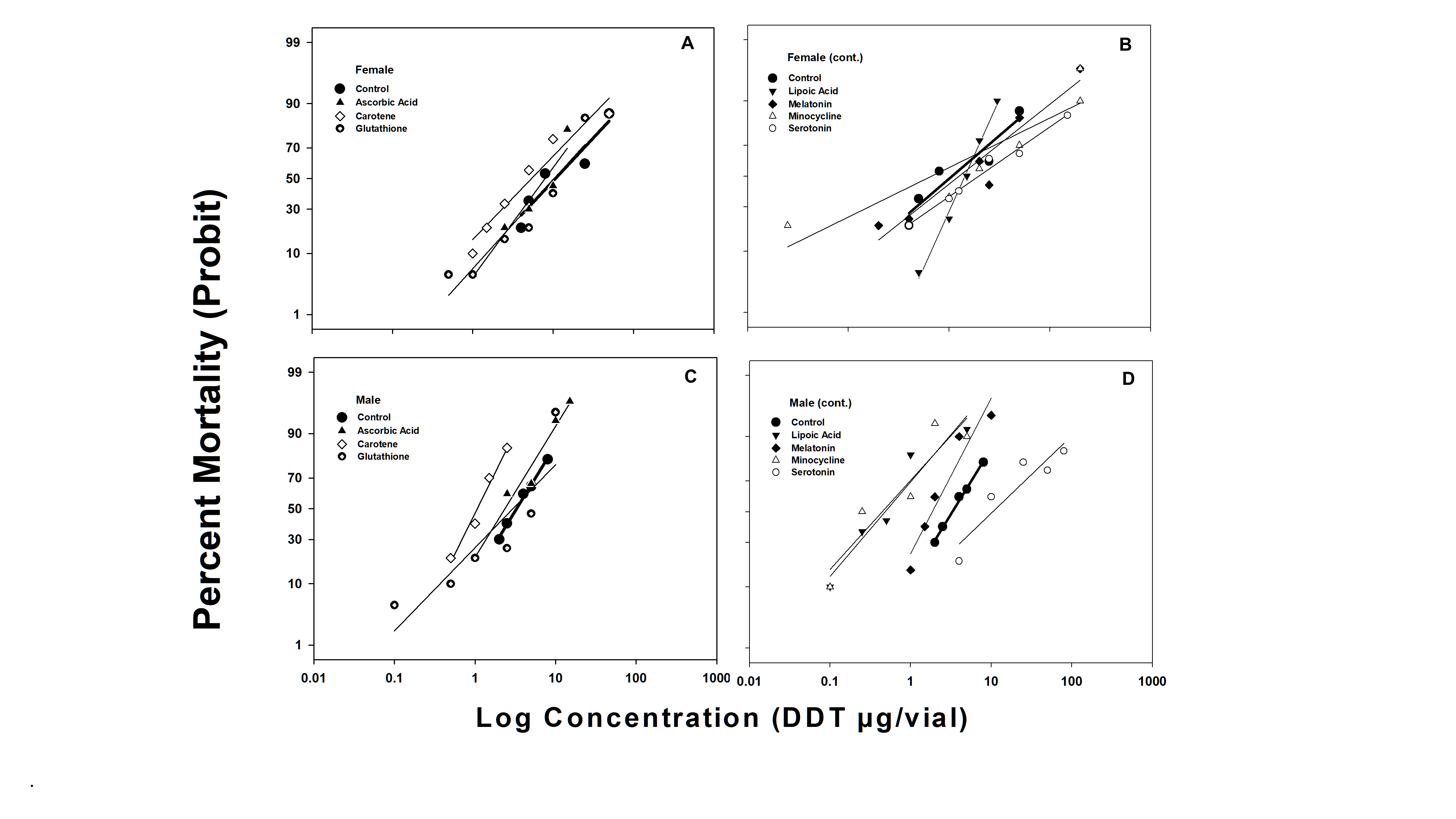

Supplement: S3 Fig — Dose response curves for DDT toxicity for females (A, B) and males (C, D) of D. melanogaster strain 91-C fed on blue diet plus antioxidants (ascorbic acid, β-carotene, glutathione, α-lipoic acid, melatonin, minocycline hydrochloride, serotonin). Adults (6–8 days old) were exposed to different doses of DDT and mortality was determined 24 h after exposure. Data were analyzed using probit analysis in SPSS (Chicago, IL, USA). For each dose, 3–4 replicates were conducted. (PNG) [file pone.0237986.s003.png]

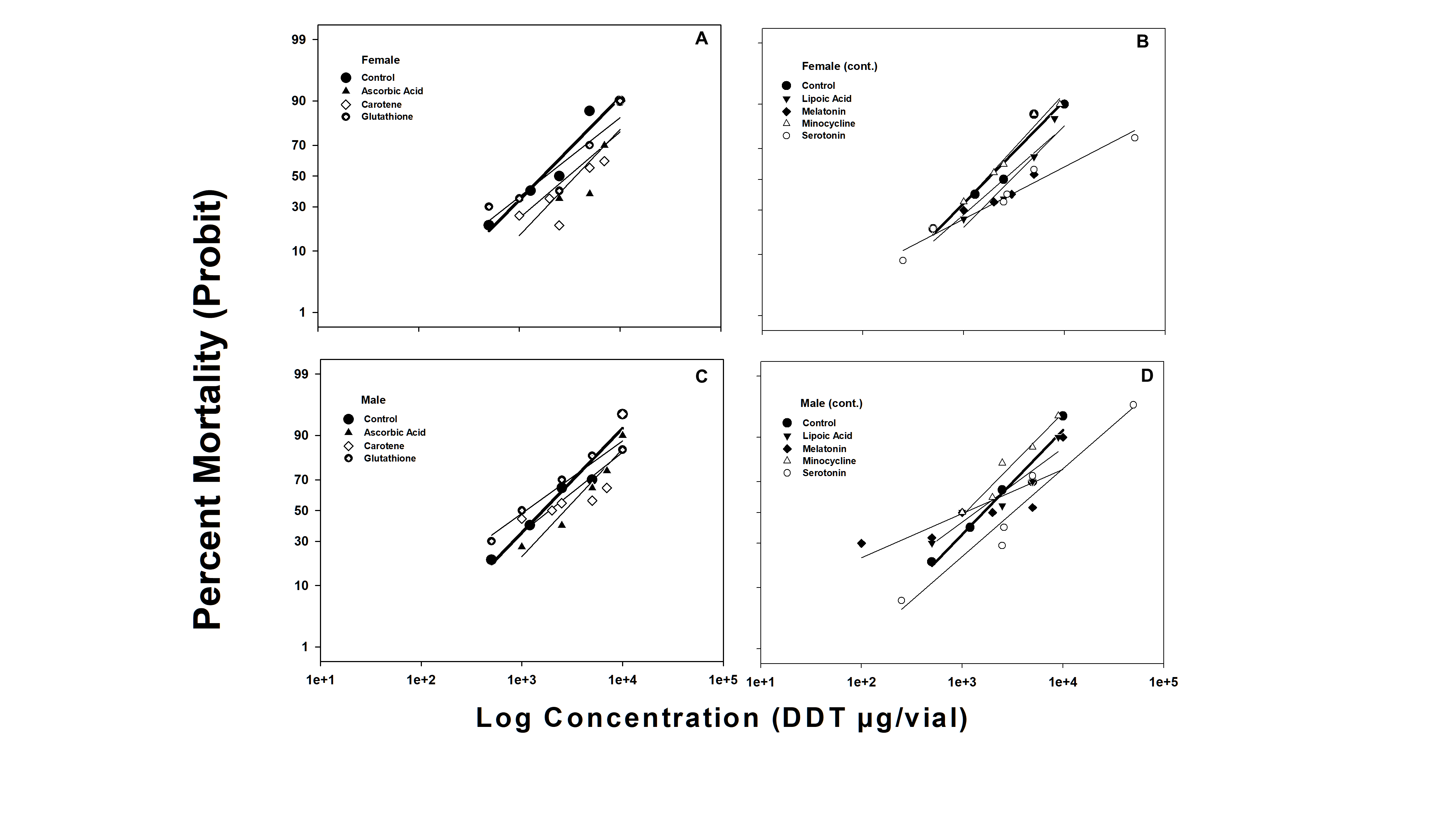

Supplement: S4 Fig — Dose response curves for DDT toxicity for females (A, B) and males (C, D) of D. melanogaster strain 91-R fed on blue diet plus antioxidants (ascorbic acid, β-carotene, glutathione, α-lipoic acid, melatonin, minocycline hydrochloride, serotonin). Adults (6–8 days old) were exposed to different doses of DDT and mortality was determined 24 h after exposure. Data were analyzed using probit analysis in SPSS (Chicago, IL, USA). For each dose, 3–4 replicates were conducted. (PNG) [file pone.0237986.s004.png]

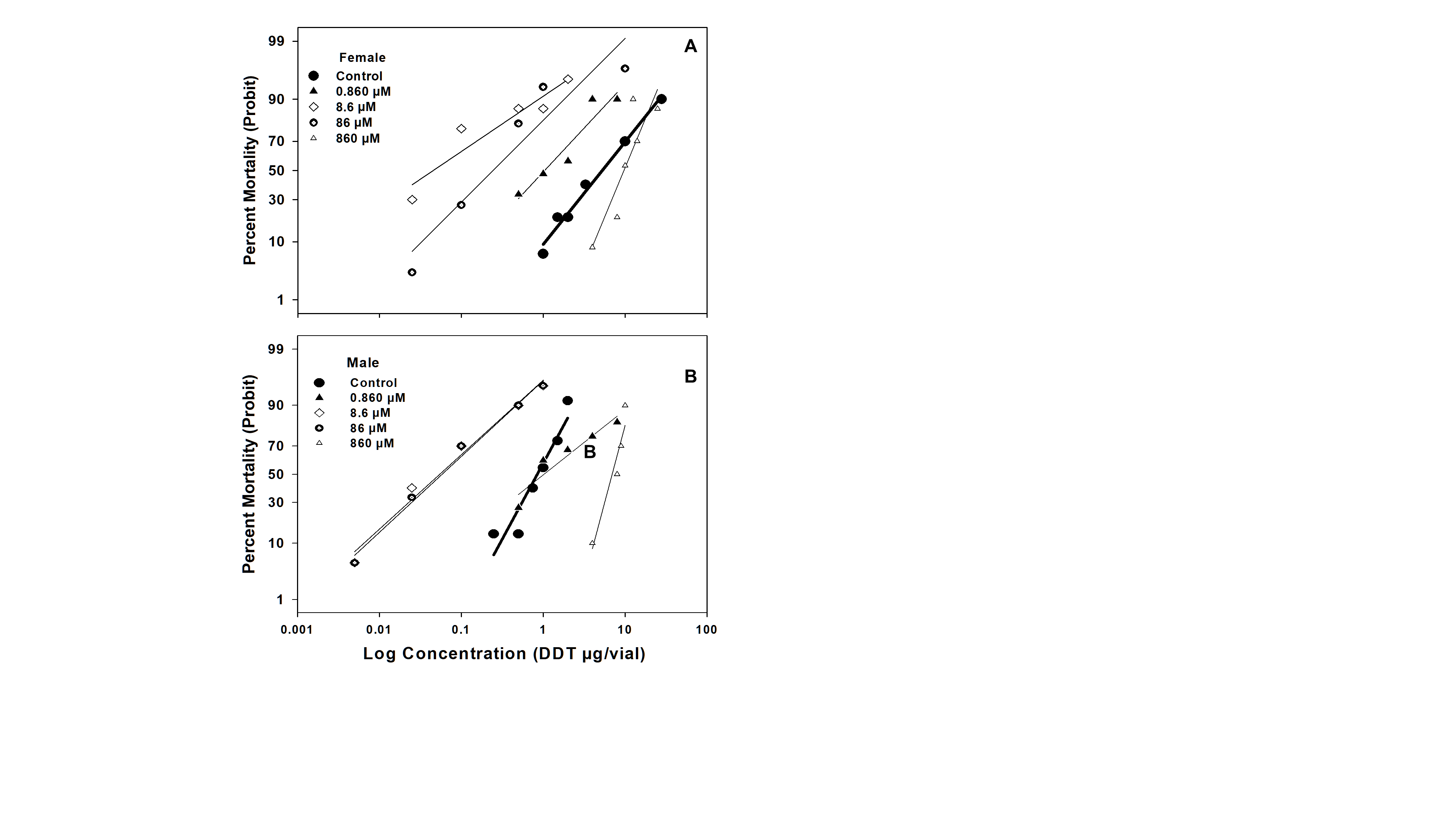

Supplement: S5 Fig — Dose response curves for DDT toxicity for females (A) and males (B) of D. melanogaster strain Canton-S fed on blue diet plus different doses of serotonin. Adults (6–8 days old) were exposed to different doses of DDT and mortality was determined 24 h after exposure. Data were analyzed using probit analysis in SPSS (Chicago, IL, USA). For each dose, 3–4 replicates were conducted. (PNG) [file pone.0237986.s005.png]
