# Supplementary material for: Dietary antioxidants impact DDT resistance in Drosophila melanogaster
Source: PLoS One. 2020 Aug 25;15(8):e0237986. doi: 10.1371/journal.pone.0237986 (PMC7447025; doi:10.1371/journal.pone.0237986)
Supplement: S1 Table — (DOCX) [file pone.0237986.s006.docx]

**S1 Table**. **LC_50_ (µg/vial DDT) values with 95% confidence limits (CL), slope, χ^2^, and resistance ratio (RR) for *D. melanogaster* *Canton-S* adult females and males fed on the indicated serotonin doses.**

| **Serotonin (µM)** | **Female** | | | | | **Male** | | | | |
| --- | --- | --- | --- | --- | --- | --- | --- | --- | --- | --- |
|  | **LC_50_**  **(95% CL)** | **Slope ±SE** | **χ^2^** | ***P*** | **RR^a^** | **LC_50_**  **(95% CL)** | **Slope ±SE** | **χ^2^** | ***P*** | **RR^a^** |
| **Control^b^** | 4.32^e^  (2.48–6.32) | 1.68±0.27 | 14.05 | 0.001 | 1.00 | 0.87^d^  (0.41–1.69) | 3.33±0.53 | 0.18 | 0.914 | 1.00 |
| **0.860** | 1.05^d^  (0.74–1.54) | 1.49±0.35 | 2.43 | 0.296 | 0.24 | 0.82^d^  (0.25–1.35) | 1.07±0.29 | 2.06 | 0.559 | 0.94 |
| **8.600** | 0.17**^c^**  (0.11–0.26) | 1.48±0.23 | 0.84 | 0.658 | 0.04 | 0.04^c^  (0.03–0.08) | 1.41±0.26 | 0.79 | 0.675 | 0.05 |
| **86.00** | 0.20**^c^**  (0.14–0.28) | 2.08±0.30 | 0.013 | 0.993 | 0.05 | 0.05^c^  (0.03–0.08) | 1.47±0.26 | 0.50 | 0.778 | 0.06 |
| **860.00^b^** | 10.25**^f^**  (6.66–13.89) | 3.58±0.47 | 11.98 | 0.007 | 2.37 | 7.39^e^  (4.45–10.81) | 6.69±1.08 | 8.70 | 0.034 | 8.49 |

^a^ Resistance ratio (RR) = LC_50_ of antioxidant / LC_50_ of Control.

^b^ Data from antioxidant comparison (Table 2).

^c,d,e, f^ Within each gender, LC_50_ values marked with different lower-case letters are significantly different based on non-overlap of 95% confidence limits.
